# Structural, Electric and Dynamic Properties of (Pyrrolidinium)_3_[Bi_2_I_9_] and (Pyrrolidinium)_3_[Sb_2_I_9_]: New Lead-Free, Organic–Inorganic Hybrids with Narrow Band Gaps

**DOI:** 10.3390/molecules28093894

**Published:** 2023-05-05

**Authors:** Magdalena Rowińska, Anna Piecha-Bisiorek, Wojciech Medycki, Piotr Durlak, Ryszard Jakubas, Anna Gagor

**Affiliations:** 1 W. Trzebiatowski Institute of Low Temperature and Structure Research Polish Academy of Science, P.O. Box 1410, 50-950 Wrocław, Poland; m.rowinska@intibs.pl; 2 Faculty of Chemistry, University of Wrocław, F. Joliot-Curie 14, 50-383 Wrocław, Poland; 3 Institute of Molecular Physics, Polish Academy of Sciences, Smoluchowskiego 17, 60-179 Poznań, Poland

**Keywords:** organic–inorganic metal halides, pyrrolidine, bismuth(III) iodides, band structure, phase transitions

## Abstract

Hybrid organic–inorganic iodides based on Bi(III) and Sb(III) provide integrated functionalities through the combination of high dielectric constants, semiconducting properties and ferroic phases. Here, we report a pyrrolidinium-based bismuth (**1**) and antimony (**2**) iodides of (NC_4_H_10_)_3_[M_2_I_9_] (M: Bi(III), Sb(III)) formula which are ferroelastic at room temperature. The narrow band gaps (~2.12 eV for **1** and 2.19 eV for **2**) and DOS calculations indicate the semiconducting characteristics of both materials. The crystal structure consists of discrete, face-sharing bioctahedra [M_2_I_9_]^3−^ and disordered pyrrolidinium amines providing charge balance and acting as spacers between inorganic moieties. At room temperature, **1** and **2** accommodate orthorhombic *Cmcm* symmetry. **1** displays a complex temperature-induced polymorphism. It is stable up to 525 K and undergoes a sequence of low-temperature phase transitions (PTs) at 221/222 K (I ↔ II) and 189/190 K (II ↔ III) and at 131 K (IV→III), associated with the ordering of pyrrolidinium cations and resulting in *Cmcm* symmetry breaking. **2** undergoes only one PT at T = 215 K. The dielectric studies disclose a relaxation process in the kilohertz frequency region, assigned to the dynamics of organic cations, described well by the Cole–Cole relation. A combination of single-crystal X-ray diffraction, synchrotron powder diffraction, spin–lattice relaxation time of ^1^H NMR, dielectric and calorimetric studies is used to determine the structural phase diagram, cation dynamics and electric properties of (NC_4_H_10_)_3_[M_2_I_9_]_._

## 1. Introduction

Organic–inorganic hybrid halide perovskites of ABX_3_ composition, based on (A: CH_3_NH_3_^+^ (MA), CH(NH_2_)_2_^+^, Cs^+^, Rb^+^, B: Pb^2+^, Sn^2+^; X: Cl^−^, Br^−^ or I^−^) have, in the past few years, risen as one of the most promising materials for solar cells applications. The most extensively studied methylammonium lead iodide and its derivatives have demonstrated impressive power conversion efficiencies of over 25% [1]. The unique properties of metal halides that make them attractive for solar cell applications are: the controllable band gap via the halide content, B metal site replacements or A site substitutions and a long diffusion length for both holes and electrons of over one micron [2]. Thus, they may work as absorbers and ambipolar charge conductors at the same time [3]. Despite the intensive development on organic–inorganic metal halides, Pb(II) is still necessary to achieve the high performance of solar cells. Even though the amounts of lead in solar cells are low, the toxicity issue appears to be a bottleneck to further development, together with the poor long-term stability. A new research field has been opened in the search for more environmentally friendly, lead-free metal halide materials [4,5]. Among them, compounds based on Bi(III) and Sb(III) halides have emerged as viable candidates for replacing lead-based compounds in photoelectrochemical cells [6]. These materials are less toxic than lead derivatives and are more resistant to atmospheric moisture with access to easy deposition processes for large-area production [7,8], making them an attractive alternative. Notable examples include Cs_3_Bi_2_X_9_, Rb_3_Bi_2_X_9_ and (MA)_3_[Bi_2_X_9_] [9,10,11]. Because of the high exciton-binding energies and significant non-radiative recombination from the defect states, the power conversion efficiency of MA_3_Bi_2_X_9_ is relatively low: ~1.6% [12]. Although, due to the small amount of research on molecular BI(III) and Sb(III) iodides, a full understanding of these materials has yet to be achieved.

It is acknowledged that efficient solar cells require an absorber material that possesses three crucial characteristics: a small band gap for efficient light absorption, the ability to separate charges and the capability to transport charges. While the optical band gap can be modified by means of chemical substitutions, the charge separation and transport properties necessitate a high dielectric constant to induce charge separation or a polar phase to facilitate charge transport. In this regard, the bismuth and antimony halides emerge as a new field for exploration. They offer high dielectric constants, as the metal ions possess 6*s*^2^ (Bi^3+^) and 5*s*^2^ (Sb^3+^) lone electron pairs and additionally may adopt phases with polar or ferroelectric ordering. Combining these properties with narrow band gaps leads to semiconducting phases with high relative permittivity or polar/ferroelectric characteristics. The presence of ferroelectric phases combined with small optical band gaps ~2 eV was confirmed for (dimethylimidazolium)_3_[Bi_2_I_9_] as well as for (formamidinium)_3_[Bi_2_I_9_] [13,14]. 

The primary structural feature of iodo-bismuthates(III) and -antimonates(III) is the MI_6_ octahedron, which serves as a fundamental building block of the crystal structure. Since Bi^3+^ and Sb^3+^ weakly bind the outer s electrons, the ions are easily polarizable. This, coupled with the weak covalency of the Bi-I and Sb-I bonds, permits a significant degree of distortion and aggregation of the MI_6_^3−^ octahedra. Diverse anionic structural patterns are generated, ranging from separate mono- or polynuclear species to higher-dimensionality (1D, 2D or 3D) substructures with a rich diversity of anionic networks [15]. The compounds with most appealing dielectric properties adopt the following chemical stoichiometries: R_5_M_2_X_11_—(0D), R_2_MX_5_—(1D), RMX_4_—(1D) and R_3_M_2_X_9_—(2D and 0D), where R represents an organic amine, M stands for Sb(III) or Bi(III) and X represents Cl, Br or I. They all were found to exhibit ferroelectric ordering when crystallized with small-sized organic cations [14]. Among the iodide derivatives, compounds with the stoichiometry R_3_M_2_I_9_ with the face-sharing bioctahedra (0D) anionic motifs are particularly noteworthy due to their potential for use in optoelectronics and solar cells, as they are exceptionally stable [16,17]. Additionally, they may exhibit multiferroic properties by combining ferroelectricity and ferroelasticity generated via numerous structural PTs [18]. 

Herein, we present a detailed physicochemical characterization of newly designed pyrrolidinium templated bismuth(III) and antimony(III) iodides. We have chosen pyrrolidinium as the counterion as it has a significant dipole moment and polarizability, which is expected to enhance the dielectric parameters, such as the dielectric constant, and improve the semiconductor properties. The small size, symmetry and possible conformational changes in these cations are also closely related to the complex molecular motions of the molecules, which are limited by temperature and may lead to a rich polymorphism in the solid state. Both compounds consist of a zero-dimensional anionic substructure, which is composed of face-sharing octahedra. By utilizing diffraction experiments on single crystals and powders; UV-Vis, dielectric and NMR spectroscopy; and DFT calculations, we have developed PT diagrams and identified molecular order–disorder processes. Furthermore, we show that both compounds have a semiconducting character and exhibit a high dielectric constant. This combination, along with their exceptional chemical stability, makes these new lead-free organic–inorganic hybrids highly promising for optoelectronic applications.

## 2. Results and Discussion

### 2.1. Thermal Properties of ***1*** and ***2***

**Figure 1:**
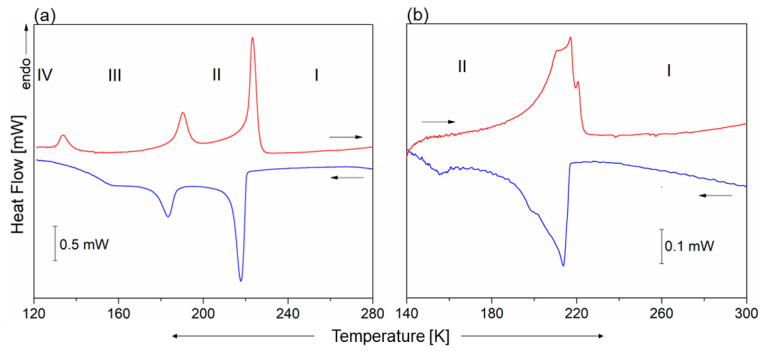
The DSC curves for (**a**) **1** and (**b**) **2** crystals measured on heating and cooling.

The DSC curves, presented in Figure 1, show three thermal anomalies in **1** that may be assigned to structural PTs: at 221/222 K (cooling/heating) from Phase I to Phase II (I→II), at 189/190 K (II→III) and 131 K (IV→III) (seen on heating). Even though the temperature hysteresis of the observed thermal anomalies is small, their sharp peak shape indicates the discontinuous PTs. The nature of the low-temperature PT (III→IV) is unclear as the thermal anomaly is only observed in the heating cycle. The transition entropies (∆S_tr_) are as follows: ∆S_tr(I→II)_ = 16 J/mol × K, ∆S_tr(II→III)_ = 6.7 J/mol × K and ∆S_tr(IV→III)_ = 4.1 J/mol × K, indicating the order–disorder character of all PTs. In turn, the antimony analog only undergoes one PT at around 215 K. This anomaly seems to be atypical, being distributed over ca. 30 K; however, it is very easily reversible. Both, the shape of this anomaly and lack of thermal hysteresis indicate its continuous character.

### 2.2. Crystal Structure of Phase I

Both **1** and **2** have a stoichiometry of R_3_M_2_I_9_, which is frequently observed in hybrids of iodoantimonates(III) and iodobismuthates(III) when they crystallize with small alkylammonium and non-substituted heteroaromatic protonated amines. The anionic component consists of face-sharing bioctahedra [M_2_I_9_]^3−^, forming a discrete (0D) substructure that interacts with the organic part through weak N-H^…^I and C-H^…^I hydrogen bonds. At high temperatures, the imidazolium (IM) [19], dimethylammonium (DMA) [20], guanidinium (G) [21], formamidynium (FA) [14] and methylammonium (MA) [22] hybrids adopt the prototypic hexagonal basic structure of *P6_3_/mmc* symmetry. In this phase, the organic cations exhibit large temperature-induced dynamical disorder. As the temperature drops, rotations are blocked, leading to structural PTs and symmetry breaking. The aristotype phase transforms to a room-temperature orthorhombic *Cmcm* in G-, IM- and DMA-based iodobismuthates(III) and iodoantimonates(III), introducing complex ferroelastic structures with *6/mmmFmmm* symmetry reduction, leading to the formation of three different twin domain states [23]. The hexagonal phase is highly disordered, with large displacement ellipsoids for metal and halide atom positions and massive disorder of the organic cations, which perform in-plane and out-of-plane rotations. The symmetry of the basic hexagonal structure is lowered by distortions of the [M_2_I_9_]^3−^ units, as well as displacements and ordering of cations. Nonetheless, the disorder of the amines endures, although to a lesser degree, even after the phase transition.

**Figure 2:**
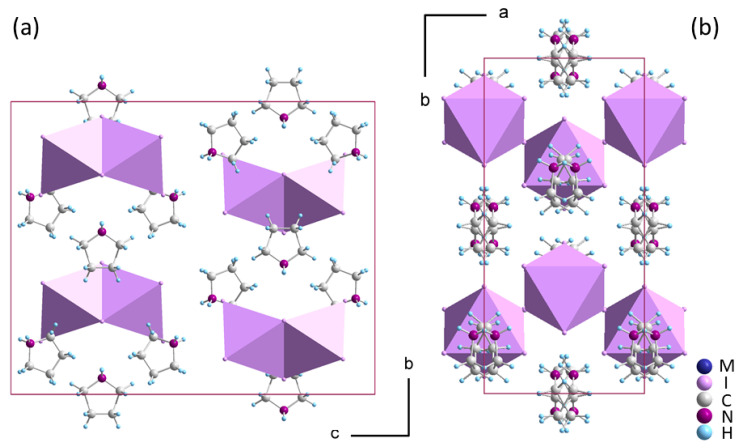
The crystal structure of the orthorhombic *Cmcm* phase of (pyrrolidinium)_3_[M_2_I_9_] (M: Sb(III) or Bi(III)) with discrete [M_2_I_9_]^3−^ units (**a**); the pseudo-hexagonal arrangement of the crystal structure and disordered pyrrolidinium amines (**b**).

**Figure 3:**
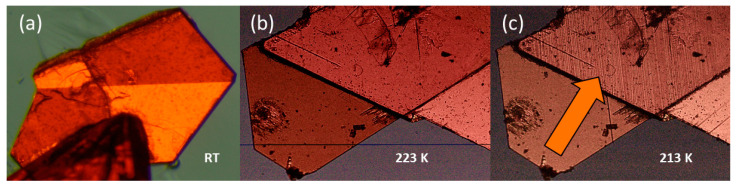
(**a**) Ferroelastic domains in **1,** at room temperature, corresponding to the *6mmmFmmm* symmetry reduction; (**b**) before and after (**c**) I→II PT; tiny parallel twin domains are formed, indicating symmetry reduction from orthorhombic to monoclinic.

All the listed compounds **1** and **2** crystallize in the orthorhombic *Cmcm* structure (see the packing in Figure 1a), which is the maximal isotropic subgroup of *P6_3_/mmc*. Similar to pyrazolium templated (PYR)_3_[M_2_I_9_] [24], **1** and **2** decompose before reaching the hexagonal aristotype. The presence of hexagonal symmetry is evident, however, through the pseudohexagonal arrangement of the crystal structure, as illustrated in Figure 2b, and existence of ferroelastic domains at ambient temperature shown in Figure 3a. 

The distinguishing feature of the orthorhombic structure is the symmetric arrangement of [M_2_I_9_]^3−^ bioctahedra of *m*2*m* symmetry. Both metal ions occupy m.. symmetry sites with near-identical bridging as well as near-identical apical Bi-I distances, being approximately 3.242 Ȧ and 2.951 Ȧ, respectively. Such a small bond distortion is one of the characteristics of the hexagonal aristo-structure rather than the orthorhombic one. The organic component is situated at two symmetry-inequivalent sites and displays significant disorder. The mirror planes in *Cmcm* split all non-aromatic pyrrolidinium cations into two equivalent sites with equal 0.5 site occupancies. This contrasts with PYR- and IM-templated hybrids, in which the planar configuration of amines allows one of the two inequivalent amines to become ordered in the *Cmcm* orthorhombic phase. The pronounced disorder of pyrrolidinium is also influenced by the potential for conformational changes in this non-aromatic amine.

The polyhedral distortion parameters collected in Table 1 show that the geometries of bioctahedral units remain constant irrespective of the cation employed in the disordered orthorhombic *Cmcm* phase. This observation suggests that the hydrogen bond interactions in this phase are feeble and do not perturb the geometry of the anionic component.

### 2.3. Low-Temperature Phases in ***1***

**Figure 4:**
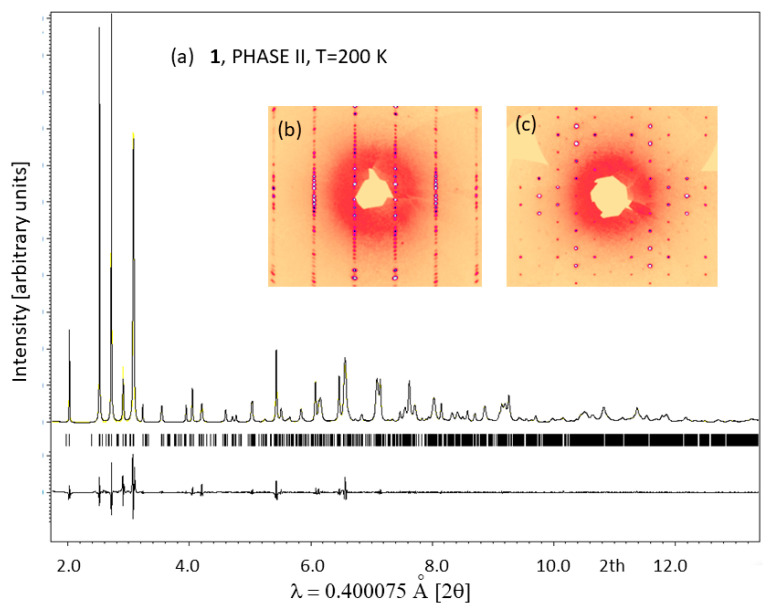
(**a**) Le Bail profile refinement results for SXRD collected at 200 K in phase II. The peaks are all indexed in the monoclinic superstructure with a = 8.7409(2) Ȧ, b = 18.1981(3) Ȧ, c = 45.3086(7) Ȧ, β = 90.306(2)° and R_p_ = 0.04 *w*R_p_ = 0.06; the insets show reciprocal space reconstructions of *h1l* (**b**) and *hk1* (**c**) planes. The positions of Bragg peaks are marked by short lines below the profile.

**Figure 5:**
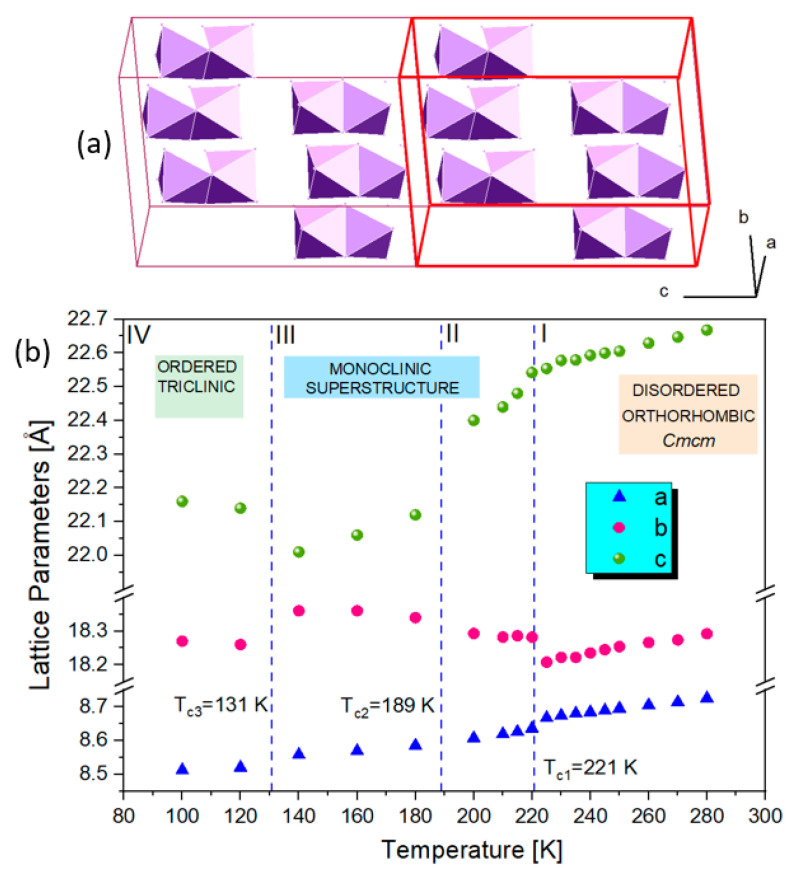
(**a**) The relation between the high-temperature (red) and low-temperature unit cells (2c superstructure); (**b**) thermal evolution of lattice parameters in **1** measured from the single-crystal X-ray diffraction. The estimated standard deviations are of the order of the marked points.

Unlike hybrids templated by aromatic ions such as GUA, IM and PYR, **1** transforms to a large-volume superstructure during the low-temperature PT. This is accompanied by further symmetry reduction, which is reflected in extra diffraction peaks from twinned domains. Figure 4 shows the Le Bail profile fit of the synchrotron X-ray data collected for **1** in phase II at 200 K. The diffracted patterns are indexed in the *a*, *b*, *2c* superstructure (see Figure 5a), which is monoclinically distorted; all diffraction patterns may be indexed in the *C2/c* space group. The single-crystal X-ray diffraction data confirm the huge unit cell with a = 8.7409(2) Å, b = 18.1981(3) Å, c = 45.3086(7) Å, β = 90.306(2)° and a volume of 7207 Å^3^. Additional Bragg peaks appear along the *c* axis, while the *a* and *b* directions remain unchanged. Moreover, the broadening and blurring of diffraction peaks in the (a,c) plane indicate twinning of the crystal due to the symmetry reduction (see the reciprocal space reconstructions for of h1l (b) and hk1 (c) planes in the inset of Figure 4). The twinning of the structure is also confirmed in the optical images taken before and after the I→II PT. In phase II, tiny parallel twin domains emerge. The development of the ferroelastic domain walls during PT (I↔II) in **1** is shown in Figure 3c. Further cooling stabilizes phase III with the unit cell analogous to phase II, indicating the symmetry changes within the monoclinic class or isostructural phase transition. Phase IV, at 90 K, reveals additional patterns that can be indexed in the triclinically distorted unit cell of II and III. The Le Bail profile fitting of phase IV is presented in supplementary information in Figure S1.

In all phases of **1**, there are small shifts and small splitting of the diffraction patterns, indicating rather insignificant reductions in the interatomic distances in this material. It is very different from low-temperature behavior of (PYR)_3_[Bi_2_I_9_], where a monoclinic distortion of the *Cmcm* orthorhombic cell leads to a substantial reduction in the crystal volume during the transition. This is because the flat aromatic PYR rings rotate by almost 90 degrees during the PT to monoclinic LT phase, causing dramatic changes in the interatomic distances between the complex anions. The unit cell collapses in the b-direction by almost 3 Å, resulting in a reduction in the distance between every other [M_2_I_9_]^3−^ from 17.2 Å to 14.5 Å at T_c_ [25]. The thermal evolution of the orthorhombic unit cell parameters of **1** is shown in Figure 5. The distortions of the crystal structure at the phase transition temperatures are reflected well by the changes in lattice parameters.

### 2.4. Electronic Properties

**Figure 6:**
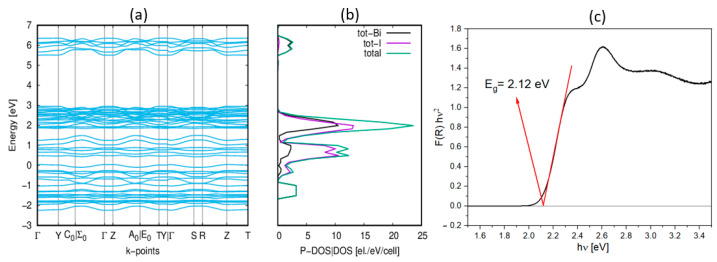
(**a**) The unified electronic band structure (EBS) of **1**; (**b**) the density of states (DOS|P-DOS) of **1** derived from the ab initio calculations; (**c**) band gap (E_g_) estimation with Tauc plots for **1**.

The semiconducting character of **1** was confirmed via *ab initio* DFT calculations. The densities of states projected onto the energy band diagram show that the highest valence states are predominantly composed of I(p) states, whereas the lowest conduction band states primarily involve bismuth p states, as shown in Figure 6. Given this information, we can infer that the electron transitions observed in the absorption spectrum are due to the ligand–metal charge transfer between I(p) and Bi(p) orbitals. The conduction band minimum and the valence band maximum are located at the same crystal momentum (k-vector) values in the Brillouin zone (Γ point), indicating a direct band gap.

The energy band gaps (E_g_) for both **1** and **2** were also determined experimentally from UV-Vis absorption spectra, using the Kubelka–Munk [26] relation. Graphical estimates of E_g_ were derived from Tauc plots (as illustrated in Figure 6c, Figures S2 and S3), yielding values of 2.12 eV and 2.19 eV for **1** and **2**, respectively. For **1,** the difference between the experimentally determined (2.12 eV) and calculated band gap value (2.5 eV) is minute, which is a testament to the accuracy of the corresponding DFT calculations. This is particularly noteworthy considering the presence of disorder in the *Cmcm* phase and the absence of designated positions of hydrogen, which did not allow for the optimization of the atomic positions for calculations. The calculated band gap of **1** is slightly larger than that of (PYR)_3_[Bi_2_I_9_] (2.2 eV) and (FA)_3_[Bi_2_I_9_] (1.85 eV). This is because larger pyrrolidine cations cause an increase in the distance between the bioctahedra. In comparison to the pyrazolium analog, the lattice parameter *b* in **1** is larger by over 1 Å, while the volume expands by over 200 Å^3^.

### 2.5. Dielectric Response 

**Figure 7:**
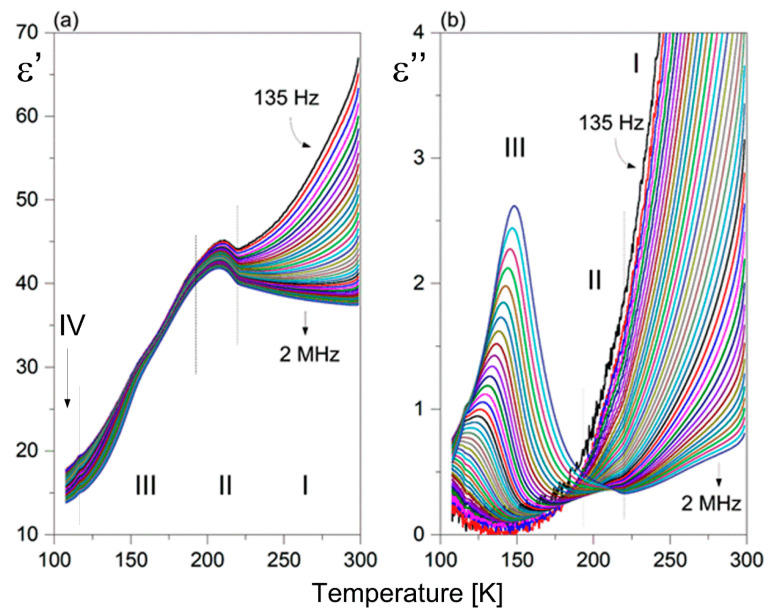
Temperature dependence of the (**a**) real (ε’) and (**b**) imaginary (ε”) part of the complex dielectric constant measured for **1** (heating cycle).

Figure 7 presents the temperature dependence of the real (a) and imaginary part (b) of the dielectric constant at various frequencies for **1**. It should be underlined that the dielectric function (ε’(T)) exhibits anomalous behavior in the temperature range covering PTs, resembling a sickle-like shape. Moreover, the maximum dielectric permittivity does not coincide with the PT temperatures. Only the higher temperature PT (I↔II) is reflected in the dielectric response, seen as a bend in the ε’(T) curve. The PT (II↔III) is not dielectrically active. It is interesting that the value of ε’ in the temperature range covering PTs clearly exceeds 40 units, which indicates enhanced electrical polarizability. Above 250 K, one can observe a strong increase in the ε’ value at lower frequencies due to an evident contribution of the conductivity phenomena. Over phase III, the ε′ displays low-frequency dispersion, and the value of ε’ is characterized by the presence of frequency-dependent maxima, as shown in Figure 7b. The maximum position shifts upward as the frequency increases. It is unexpected that the value of dielectric losses increases markedly with increasing frequency with temperature. Such a dielectric response is characteristic of glass-like dielectric properties over phase III [27,28].

**Figure 8:**
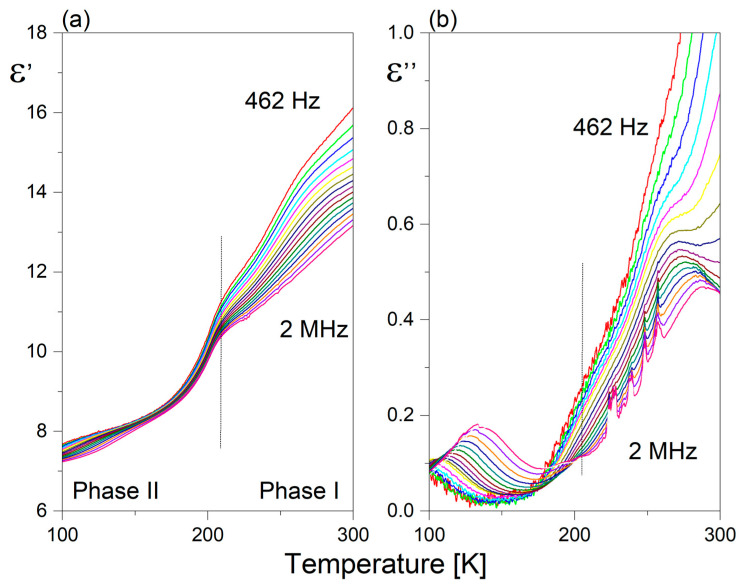
Temperature dependence of the (**a**) real (ε’) and (**b**) imaginary (ε”) part of the complex dielectric constant measured for **2** (heating cycle).

The temperature behavior of the real (ε’) and imaginary parts (ε”) of the dielectric constant at selected frequencies of **2** is shown in Figure 8a,b, respectively. It seems that the dielectric function of **2** differs from that observed for **1**. PT (I↔ II) is dielectrically active, but the values of the static dielectric constant are nearly three times lower compared to ε’_o_ in **1**. Correspondingly, the lowest frequency dielectric relaxator observed over phase II for **2** is distinctly weaker than that in **1**, since its dielectric increment is close to one order less (Δε’ ~0.2). Nevertheless, it should be underlined that the lower-temperature phases of both pyrrolidinium analogs are characterized by a presence of glass-like properties.

### 2.6. Cations Dynamics in ***1*** from Proton Magnetic Resonance (^1^H NMR)

**Figure 9:**
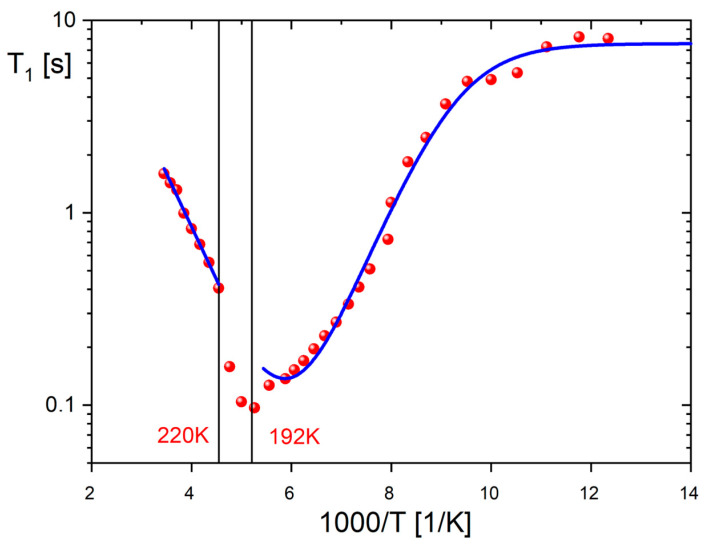
^1^H spin–lattice relaxation time T_1_ versus temperature for **1** at 25 MHz and theoretical predictions (dotted lines) obtained from numerical fitting.

Figure 9 illustrates the temperature-dependent behavior of the ^1^H NMR spin–lattice relaxation time (*T*_1_) for **1**, ranging from 81 K to 290 K. The PTs occurring at 189 K and 221 K have a significant impact on the dynamics of the cation substructure. However, the wide minimum over phase III is not well-defined, as it is superimposed by two structural PTs. Moreover, the observed flattening of relaxation times at low temperatures can be attributed to the interaction between protons and the close quadrupole iodine atoms. The relaxation of the studied compound is a result of two relaxation mechanisms: the first one corresponds to the interaction between hydrogen and quadrupole nuclei, responsible for the low-temperature flattening, while the second one is related to classical ^1^H–^1^H dipole–dipole interactions, leading to the relaxation minimum at higher temperatures. Thus, the *T_1_* relaxation time dependence was fitted using the sum of two components throughout the temperature range [7,8]:

$$\frac{1}{T_{1}}=C_{HH}\left[\frac{\tau_{c}}{1+\omega_{H}^{2}\tau_{ci}^{2}}+\frac{4\tau_{c}}{1+4\omega_{H}^{2}\tau_{c}^{2}}\right]+C_{HQ}\left[\frac{\tau_{c,Q}}{1+(\omega_{H}-\omega_{Q}{)}^{2}\tau_{c,Q}^{2}}+\frac{3\tau_{c,Q}}{1+4\omega_{H}^{2}\tau_{c,Q}^{2}}+\frac{6\tau_{c,Q}}{1+(\omega_{H}+\omega_{Q}{)}^{2}\tau_{c,Q}^{2}}\right]$$

where *C_HH_* is the relaxation constant of ^1^H–^1^H dipole–dipole interactions, *C_HQ_* is the relaxation constant of quadrupole interaction and *ω_Q_* is the Larmor frequency of the halogen nuclei, iodine. The correlation time, *τ_c,Q_*_,_ is defined as: *τ_c;Q_*^−1^_=_ *τ_c_*^−1^ + *R_Q_*, where *R_Q_* represents the quadrupolar spin–lattice rates of the dominating component, which, in the present case, is halogen nuclei of ^127^I. After the numerical fitting to the experimental data (red line in Figure 9), the following parameters were found: *E_a_* = 11.85 kJ/mol, *τ_c_* = 6.26 · 10^−13^ s, *C_HH_* = 2.71 · 10^8^ s^−2^, *C_HQ_* = 5.6 · 10^8^ s^−2^ and *R_Q_*^−1^ = 1.91 · 10^−7^ s. Here, the quadrupole relaxation has been assumed as temperature-independent. Contributing to the effective modulations of the ^1^H–^127^I dipole–dipole coupling causes the ^1^H relaxation times to be constant (the plateau in Figure 9) despite a very long correlation time *τ_c_* at low temperatures. As the temperature rises above the intermediate phase II (192–220 K), the measured relaxation times change linearly with a slope of relaxation minimum and an activation energy of approximately 10.5 kJ/mol. 

As the temperature of the system increases, the pyrrolidinium cation may initiate various types of molecular motions that can impact the observed proton–proton dipole interaction in NMR experiments [1,2,3,4,5,6]:

(i). Small-angle rotations of the pentagonal ring;
(ii). Changes of the pyrrolidinium ring conformation (twisted/envelope conformation);
(iii). Rotation about the pseudo-C_5_ axis perpendicular to the pyrrolidine ring;
(iv). Isotropic rotation and possibly cationic self-diffusion/rotational diffusion at the highest temperatures.

The aforementioned motion type (iv) is not applicable in our case. From the obtained *M*_2_ values, it can be concluded that the cationic network remains rigid throughout phase III, up to 192 K (where *M*_2_ approaches 18 units), indicating a possible motion of type (i). As the temperature increases into the intermediate phase II, there is a gradual release of the type motion (ii) of the pyrrolidinium ring. Additionally, as the *M*_2_ value drops to around 5.5–6 units in phase I, a gradual release of the C_5_-type rotation is postulated. The spin–lattice relaxation time measurements (depicted in Figure 9) indicate that the relaxation process above 220 K is dominated by pseudo-C_5_ jumps of the cations. This is further confirmed by the activation energy of approximately 10 kJ/mol, which is characteristic of the reorientation of the pyrrolidinium ring in various compounds [29,30,31,32,33,34]. The visible temperature anomalies on the *T*_1_(1/T) curve around 192 and 220 K clearly indicate the order–disorder mechanism of PTs, owing to the drastic change in the motional state of the cations. Structural PTs are also evidenced on the temperature dependencies of the second moment *M*_2_ of the ^1^H NMR line (see Figure S4). Near the PT at 189 K, a reduction in the second moment from 18 × 10^−8^ T^2^ to 12 × 10^−8^ T^2^ confirms a significant change in the dynamics of the pyrrolidinium cations.

## 3. Experimental Section

### 3.1. Synthesis

Commercially sourced materials, including pyrrolidine (≥99%), BiI_3_ (99.98%), SbI_3_ (98%) and HI (57%), were utilized without further purification to synthesize the pyrrolidinium analogs (C_4_NH_10_)_3_[Bi_2_I_9_] (**1**) and (C_4_NH_10_)_3_[Sb_2_I_9_] (**2**). The crystals were grown by gradually evaporating a concentrated HI solution containing a 3:2 ratio of pyrrolidine and BiI_3_ or SbI_3_. The resulting salts were recrystallized twice from either methanol or acetonitrile (CH_3_CN) solutions. The elemental analysis confirmed their compositions: **1**: C: 8.16% (*theor*. 8.11%), N: 2.40% (*theor*. 2.37%) and H: 1.72% (*theor*. 1.70%); and **2**: C: 8.97% (*theor*. 9.00%), N: 2.65% (*theor*. 2.62%) and H: 1.88% (*theor*. 1.89%). The single crystals were grown at room temperature from an ethanol/acetonitrile solution.

### 3.2. Single-Crystal X-ray Diffraction

X-ray diffraction experiments were conducted using an Xcalibur four-circle diffractometer (Oxford Diffraction) equipped with an Atlas CCD detector and graphite-monochromated Mo Kα radiation. The multi-scan method in CrysAlis PRO 1.171.39.46 (Rigaku Oxford Diffraction, 2018) was used to correct for absorption, and the SCALE3 ABSPACK scaling algorithm was utilized to apply empirical absorption correction using spherical harmonics. For both compounds, the H atom parameters were constrained. Crystal structures were solved in Olex2 1.5 [35] using SHELXT [36] and refined using SHELXL [37]. The experiments were conducted at 240 K for **1** and 230 K for **2**. Detailed experimental and geometric parameters can be found in Tables S1–S4 in the supplementary information file. The concise structural details are as follows: (Pyrrolidinium)_3_[Bi_2_I_9_] (I, 230 K): orthorhombic, *Cmcm*, *a* = 8.6726(3) Å, *b* = 18.1683(8) Å, *c* = 22.5902(9) Å, β = 90°; *V* = 3559.5(2) Å^3^, *Z* = 4, *R*_1_ = 0.052, *wR*_2_ = 0.105, *S* = 1.02; (Pyrrolidinium)_3_[Sb_2_I_9_] (I, 240 K): orthorhombic, *Cmcm*, *a* = 8.5167(5) Å, *b* = 18.2142(11) Å, *c* = 22.6087(19) Å, β = 90°; *V* = 3507.2 (4) Å^3^, *Z* = 4, *R*_1_ = 0.053, *wR*_2_ = 0.130, *S* = 1.07. The crystal structures of **1** and **2** have been deposited in CCDC with numbers: 2249176 for **1**, 230 K, 2249177 for **1** at room temperature and 2249178 for **2** at 240 K. 

### 3.3. Synchrotron Powder Diffraction

Synchrotron data were collected for **1** at the European Synchrotron Radiation Facility (ESRF, Grenoble, France) at high-resolution powder diffraction beamline ID 22. The sample was measured in a glass capillary in transmission mode with λ = 0.400075Ȧ.

### 3.4. Optical Measurements

The ferroelastic domain structures were observed by means of an Olympus BX53 optical polarization microscope combined with a LINKAM THM-600 heating/cooling stage. The temperature was stabilized to within 0.1 K.

### 3.5. Thermal Analysis

DSC measurements were performed by heating and cooling the polycrystalline samples in the temperature range of 100–300 K with a ramp rate of 10 K min^−1^ using a Metler Toledo DSC 3 instrument. The TGA/DSC measurements were performed on a TGA-DSC 3 instrument between 290 and 900 K with a ramp rate of 5 K min^−1^. The scan was performed in flowing nitrogen (flow rate: 1 dm^3^ h^−1^).

### 3.6. Electrical Measurements

Electrical measurements were performed on the pressed pellets (for both analogs) with the following geometrical parameters: *S* = 20 mm^2^ and *d* = 0.7–0.8 mm. The complex dielectric permittivity was measured between 100 and 300 K using an Agilent E4980A Precision LCR Meter in the frequency range of 135 Hz–2 MHz. The temperature was stabilized and controlled using an INSTEC STC200. The electric measurements were carried out in a controlled nitrogen atmosphere. The surfaces of the crystal were coated with silver-conductive paint.

### 3.7. NMR 

NMR measurements were conducted for **1** using an ELLAB TEL-Atomic PS 15 at a frequency of 25 MHz. The spin–lattice relaxation times (*T*_1_) were determined using a saturation sequence consisting of a π/2 pulse, a variable time interval τ, and a reading π/2 pulse. The magnetization was found to recover exponentially within the experimental error at all temperatures. The proton NMR second moment was assessed with an ELLAB CW spectrometer capable of wide-line detection at a frequency of 26.8 MHz. Second moment values were computed by numerically integrating the first derivative of an absorption line and adjusting for finite modulation amplitude. The sample temperature, which could be controlled down to liquid nitrogen, was regulated by a UNIPAN 660 temperature controller equipped with a Pt 100 sensor that provided long-term temperature stability of better than 1 K. All measurements were taken on a heated sample. The *T*_1_ measurement errors were estimated to be approximately 5%. The powder sample was first evacuated at room temperature and then sealed under a vacuum in a glass ampule.

### 3.8. Calculation Details

Total energy calculations were performed for **1** using *ab initio* Density Functional Theory (DFT), as implemented in the CRYSTAL17 [38,39] package, designed for use in modeling crystalline solids, and we employed the London-type empirical correction in the (D3) variant for dispersion interactions, as proposed by Grimme [40,41,42,43], including three-body dispersion contributions with fast analytical gradients together with the vibrational harmonic frequency calculations. The structural data (starting geometry) were taken from the crystal structure of **1** from the present study. The periodic *ab initio* calculations were performed utilizing the DFT-D3 methods with the range-separated (short-range-corrected) hybrid functional and the screened-Coulomb PBE functional combined with PBE correlation: HSE06-D3 [44,45] with the two shrinking factors 16′,16′) to generate a commensurate grid of k-points in reciprocal space, following the Monkhorst–Pack [46] net method. All quantum-mechanical condensed matter simulations, including single-point energy, the electronic band structure (EBS) and the density of states (DOS) were carried out with the modified basis sets coupled with the full-relativistic and scalar-relativistic effective core potentials (ECPs) for bismuth and iodine atoms, respectively. For the bismuth atom, we have used the Bi_ECP60MFD_s4411p411d411 basis set proposed by Heifets [47,48] for the calculation, and for the iodine atom, we have used the I_HAYWLC-31G* modified basis set coupled with (HAYWLC) the Hay and Wadt [49,50] large-core ECP. The electronic band structure was generated according to the procedure in the CRYSTAL17 program, and the SeeK-path [51] program was used to determine the k-points along a path within the first Brillouin zone, including the surface in reciprocal space. The EBS and DOS data from calculations were visualized as *a posteriori* in the Gnuplot [52] program.

## 4. Conclusions

In summary, we have synthesized new, lead-free hybrid halide semiconductors based on pyrrolidinium cations, namely (C_4_NH_10_)_3_[Bi_2_I_9_] (**1**) and (C_4_NH_10_)_3_[Sb_2_I_9_] (**2**), which undergo structural PTs: (**1**) 221/222 K (I↔II), 189/190 K (II↔III) and at 131 K (IV→III); (**2**) at 215 (I↔II). These transitions are accompanied by the order−disorder rearrangements of organic cations and the deformation of anionic [M_2_I_9_]^3-^ bioctahedra. Single-crystal X-ray and powder synchrotron diffraction data confirm symmetry reduction in **1** from *Cmcm* to a large monoclinic superstructure at PT (I↔II). The phase transitions may be summarized as follows: 
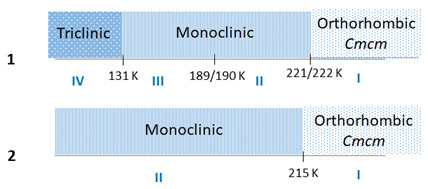


Compound **1** shows remarkable dielectric properties in phase I, particularly around the PT(I↔II) with a maximum permittivity (ε’_max_) of approximately 45. Furthermore, a low-frequency dielectric relaxation process observed in the lowest-temperature phases of **1** (phase IV) and **2** (phase II) exhibits glass-like behavior. Both compounds exhibit ferroelastic properties in all phases. The ^1^H NMR spin–lattice relaxation time and second moment M_2_ studies disclose distinct temperature anomalies around 190 and 222 K, confirming the order–disorder mechanism of PTs resulting from the significant change in cation motion. Both **1** and **2** possess narrow band gaps of 2.12 eV and 2.19 eV, respectively. The electrical properties of these two new pyrrolidinium analogs of lead-free organic–inorganic hybrids are not restricted by the dimensionality of the connections between the metal halide octahedra, unlike lead halide perovskites. Although **1** and **2** have 0D discrete anionic components, their energy band gaps are comparable to 3D lead halides.

## Figures and Tables

**Table 1:** Polyhedral distortion parameters for pyrazolium- (PYR), imidazolium- (IM), and pyrrolidinium-based (**1** and **2**) iodoantimonates(III) and iodobismuthates(III).

	(PYR)_3_[Bi_2_I_9_] [24]	(PYR)_3_[Sb_2_I_9_] [24]	(IM)_3_[Bi_2_I_9_] [19]	(IM)_3_[Sb_2_I_9_] [19]	1 (This Work)	2 (This Work)
Average bond length (Å)	3.105	3.061	3.101	3.065	3.095	3.045
Polyhedral volume (Å^3^)	39.51	37.89^1^	39.36	38.05	39.17	37.30
Distortion index (bond length) *	0.042	0.049	0.049	0.057	0.047	0.060
Bond angle variance (deg.^2^)	23.98	21.82	22.85	19.38	25.08	20.79

* Calculated in Vesta, Ver 3.4.3 [24].

## Data Availability

Experimental data are available at 10.5281/zenodo.7766261 Crystal structures have been deposited in CCDC with numbers 2249176, 2249177 and 2249178.

## Supplementary Materials

The following supporting information can be downloaded at: https://www.mdpi.com/article/10.3390/molecules28093894/s1, Figure S1: Le Bail profile refinement results for SXRD data collected for (pyrrolidinium)3[Bi2I9] at 90 K; Figure S2: Diffuse reflectance spectra of 1 and 2; Figure S3: Band gap (Eg) estimation for 2; Figure S4: M2 Temperature dependence of second moment of 1H NMR line of 1; Tables S1–S4: The experimental tables for single-crystal X-ray diffraction and selected geometric parameters of the crystal structure for 1 and 2 [53,54].

## Sample Availability

Samples of the compounds **1** and **2** are available from the authors.
