# Supplementary material for: Structural, Electric and Dynamic Properties of (Pyrrolidinium)3[Bi2I9] and (Pyrrolidinium)3[Sb2I9]: New Lead-Free, Organic–Inorganic Hybrids with Narrow Band Gaps
Source: Molecules. 2023 May 5;28(9):3894. doi: 10.3390/molecules28093894 (PMC10180494; doi:10.3390/molecules28093894)
Supplement: Supplementary file 1 [file molecules-28-03894-s001.zip › molecules-2334056-supplementary.pdf]

Electronic Supporting Information for:

# Structural, electric and dynamic properties of [pyrrolidinium]$_3$Bi$_2$I$_9$ and [pyrrolidinium]$_3$Sb$_2$I$_9$; new lead-free organic-inorganic hybrids with narrow band gaps

Authors: M. Rowińska[1], A. Piecha[2], W. Medycki[3], Piotr Durlak[2], R. Jakubas[2], A. Gągor[1*]

[1] *W. Trzebiatowski Institute of Low Temperature and Structure Research Polish Academy of Science, P.O. Box 1410, Wrocław, 50-950, Poland*

[1] *Faculty of Chemistry, University of Wrocław, F. Joliot-Curie 14, Wrocław, 50-383, Poland*

[3] *Institute of Molecular Physics, Polish Academy of Sciences, Smoluchowskiego 17, Poznań, 60-179, Poland*

* - Corresponding Author; e-mail: a.gagor@intibs.pl

## Table S1. Experimental details

For all structures: crystal system orthorhombic, space group *Cmcm*, $Z = 4$. Experiments were carried out with Mo *K*a radiation using a Xcalibur, Atlas. Absorption was corrected for by multi-scan methods, *CrysAlis PRO* 1.171.38.43 (Rigaku Oxford Diffraction, 2015) Empirical absorption correction using spherical harmonics, implemented in SCALE3 ABSPACK scaling algorithm was implemented. H-atom parameters were constrained.

| | **(pyrrolidinium)$_3$[Bi$_2$I$_9$]** | **(pyrrolidinium)$_3$[Sb$_2$I$_9$]** |
|---|---|---|
| ***Crystal data*** | | |
| Chemical formula | $Bi_2I_9 \cdot 2(C_4H_{9.5}N) \cdot C_4H_{10}N$ | $I_9Sb_2 \cdot 3(C_4H_{10}N)$ |
| $M_r$ | 1776.45 | 1601.99 |
| Temperature (K) | 230 | 240 |
| *a*, *b*, *c* (Å) | 8.6726 (3), 18.1683 (8), 22.5902 (9) | 8.5167 (5), 18.2142 (11), 22.6087 (19) |
| $V$ (Å$^3$) | 3559.5 (2) | 3507.2 (4) |
| $\mu$ (mm$^{-1}$) | 17.69 | 9.47 |
| Crystal size (mm) | 0.33 × 0.21 × 0.18 | 0.25 × 0.18 × 0.07 |
| ***Data collection*** | | |
| $T_{min}$, $T_{max}$ | 0.370, 1.000 | 0.447, 1.000 |
| No. of measured, independent and observed [$I > 2\sigma(I)$] reflections | 15444, 2437, 1484 | 17033, 2402, 1685 |
| $R_{int}$ | 0.051 | 0.044 |
| $(\sin \theta/\lambda)_{max}$ (Å$^{-1}$) | 0.690 | 0.687 |
| ***Refinement*** | | |
| $R[F^2 > 2\sigma(F^2)]$, $wR(F^2)$, $S$ | 0.052, 0.105, 1.02 | 0.053, 0.130, 1.07 |
| No. of reflections | 2437 | 2402 |
| No. of parameters | 59 | 63 |
| No. of restraints | 6 | 4 |
| | $w = 1/[\sigma^2(F_o^2) + (0.029P)^2 + 57.7983P]$ where $P = (F_o^2 + 2F_c^2)/3$ | $w = 1/[\sigma^2(F_o^2) + (0.0413P)^2 + 60.7163P]$ where $P = (F_o^2 + 2F_c^2)/3$ |
| $\Delta\rho_{max}$, $\Delta\rho_{min}$ (e Å$^{-3}$) | 0.93, -0.98 | 1.17, -1.15 |

Computer programs: *CrysAlis PRO* 1.171.38.43 (Rigaku OD, 2015), *SHELXT* 2018/2 (Sheldrick, 2018), *SHELXL* 2018/3 (Sheldrick, 2015), Olex2 1.5 (Dolomanov *et al.*, 2009).

**Table S2. Selected geometric parameters (Å, º)**

| (pyrrolidinium)$_3$[Bi$_2$I$_9$] 230 K | | (pyrrolidinium)$_3$[Sb$_2$I$_9$] 240 K | |
|---|---|---|---|
| Bi1—I1$^{i}$ | 3.2429 (9) | Sb1—I2 | 3.2375 (12) |
| Bi1—I1 | 3.2429 (9) | Sb1—I3 | 3.2245 (9) |
| Bi1—I2 | 3.2458 (12) | Sb1—I3$^{i}$ | 3.2245 (9) |
| Bi1—I3 | 2.9498 (9) | Sb1—I4 | 2.8600 (9) |
| Bi1—I3$^{ii}$ | 2.9498 (9) | Sb1—I4$^{ii}$ | 2.8601 (9) |
| Bi1—I4 | 2.9505 (14) | Sb1—I5 | 2.8623 (14) |
| | | | |
| I1—Bi1—I1$^{i}$ | 83.63 (4) | I3$^{i}$—Sb1—I2 | 82.40 (3) |
| I1$^{i}$—Bi1—I2 | 81.89 (2) | I3—Sb1—I2 | 82.40 (3) |
| I1—Bi1—I2 | 81.89 (2) | I3—Sb1—I3$^{i}$ | 83.92 (3) |
| I3—Bi1—I1$^{i}$ | 89.95 (3) | I4$^{ii}$—Sb1—I2 | 92.36 (3) |
| I3$^{ii}$—Bi1—I1 | 89.95 (3) | I4—Sb1—I2 | 92.36 (3) |
| I3$^{ii}$—Bi1—I1$^{i}$ | 171.66 (3) | I4—Sb1—I3$^{i}$ | 89.67 (2) |
| I3—Bi1—I1 | 171.67 (3) | I4$^{ii}$—Sb1—I3 | 89.67 (2) |
| I3—Bi1—I2 | 91.97 (3) | I4—Sb1—I3 | 172.19 (3) |
| I3$^{ii}$—Bi1—I2 | 91.97 (3) | I4$^{ii}$—Sb1—I3$^{i}$ | 172.19 (3) |
| I3$^{ii}$—Bi1—I3 | 95.92 (5) | I4—Sb1—I4$^{ii}$ | 96.35 (5) |
| I3$^{ii}$—Bi1—I4 | 91.58 (3) | I4$^{ii}$—Sb1—I5 | 91.73 (3) |
| I3—Bi1—I4 | 91.58 (3) | I4—Sb1—I5 | 91.73 (3) |
| I4—Bi1—I1$^{i}$ | 94.18 (3) | I5—Sb1—I2 | 173.86 (4) |
| I4—Bi1—I1 | 94.18 (3) | I5—Sb1—I3$^{i}$ | 93.05 (3) |
| I4—Bi1—I2 | 174.70 (4) | I5—Sb1—I3 | 93.05 (3) |
| Bi1—I1—Bi1$^{iii}$ | 80.89 (3) | Sb1$^{iii}$—I2—Sb1 | 80.01 (4) |
| Bi1—I2—Bi1$^{iii}$ | 80.80 (4) | Sb1$^{iii}$—I3—Sb1 | 80.40 (3) |

Symmetry code(s): (i) -*x*+1, *y*, -*z*+3/2; (ii) -*x*+1, *y*, *z*; (iii) *x*, *y*, -*z*+3/2.

## Table S3. Experimental details 1 at rt

| | $(pyrrolidinium)_3[Bi_2I_9]$ RoomTemperature |
|---|---|
| ***Crystal data*** | |
| Chemical formula | $Bi_2I_9{\cdot}1.5(C_8H_{20}N_2)$ |
| $M_r$ | 1776.45 |
| Crystal system, space group | Orthorhombic, *Cmcm* |
| Temperature (K) | 298 |
| *a*, *b*, *c* (Å) | 8.7266 (3), 18.1985 (8), 22.6661 (11) |
| *V* (Å$^3$) | 3599.6 (3) |
| *Z* | 4 |
| Radiation type | Mo *K*α |
| μ (mm$^{-1}$) | 17.49 |
| Crystal size (mm) | 0.22 × 0.18 × 0.07 |
| ***Data collection*** | |
| Diffractometer | Xcalibur, Atlas |
| $T_{min}$, $T_{max}$ | 0.135, 1.000 |
| No. of measured, independent and observed [$I > 2\sigma(I)$] reflections | 36050, 2014, 1621 |
| $R_{int}$ | 0.046 |
| $(\sin \theta/\lambda)_{max}$ (Å$^{-1}$) | 0.625 |
| ***Refinement*** | |
| $R[F^2 > 2\sigma(F^2)]$, $wR(F^2)$, $S$ | 0.047, 0.114, 1.17 |
| No. of reflections | 2014 |
| No. of parameters | 59 |
| No. of restraints | 5 |
| H-atom treatment | H-atom parameters constrained |
| | $w = 1/[\sigma^2(F_o^2) + (0.0333P)^2 + 63.0922P]$ where $P = (F_o^2 + 2F_c^2)/3$ |
| $\Delta\rho_{max}$, $\Delta\rho_{min}$ (e Å$^{-3}$) | 1.11, -1.06 |

Computer programs: *CrysAlis PRO* 1.171.38.43 (Rigaku OD, 2015), *SHELXL* 2018/3 (Sheldrick, 2015), Olex2 1.5 (Dolomanov *et al.*, 2009).

## Table S4. Selected geometric parameters of 1 (Å, º)

| $(pyrrolidinium)_3[Bi_2I_9]$ _room_temperature | | | |
|---|---|---|---|
| Bi1—I2 | 3.2413 (13) | Bi1—I4$^{ii}$ | 2.9513 (10) |
| Bi1—I3 | 3.2417 (10) | Bi1—I4 | 2.9513 (10) |
| Bi1—I3$^{i}$ | 3.2417 (10) | Bi1—I5 | 2.9517 (15) |
| | | | |
| I2—Bi1—I3 | 81.48 (3) | I4$^{ii}$—Bi1—I4 | 95.90 (5) |
| I2—Bi1—I3$^{i}$ | 81.48 (3) | I4$^{ii}$—Bi1—I5 | 91.67 (3) |
| I3—Bi1—I3$^{i}$ | 83.46 (4) | I4—Bi1—I5 | 91.67 (3) |
| I4—Bi1—I2 | 92.03 (3) | I5—Bi1—I2 | 174.48 (4) |
| I4$^{ii}$—Bi1—I2 | 92.03 (3) | I5—Bi1—I3 | 94.42 (4) |
| I4$^{ii}$—Bi1—I3$^{i}$ | 171.39 (3) | I5—Bi1—I3$^{i}$ | 94.42 (4) |

| | | | |
|---|---|---|---|
| I4[ii]—Bi1—I3 | 90.01 (3) | Bi1—I2—Bi1[iii] | 81.31 (4) |
| I4—Bi1—I3 | 171.39 (3) | Bi1[iii]—I3—Bi1 | 81.30 (3) |
| I4—Bi1—I3[i] | 90.01 (3) | | |

Symmetry code(s): (i) -*x*+1, *y*, -*z*+3/2; (ii) -*x*+1, *y*, *z*; (iii) *x*, *y*, -*z*+3/2.

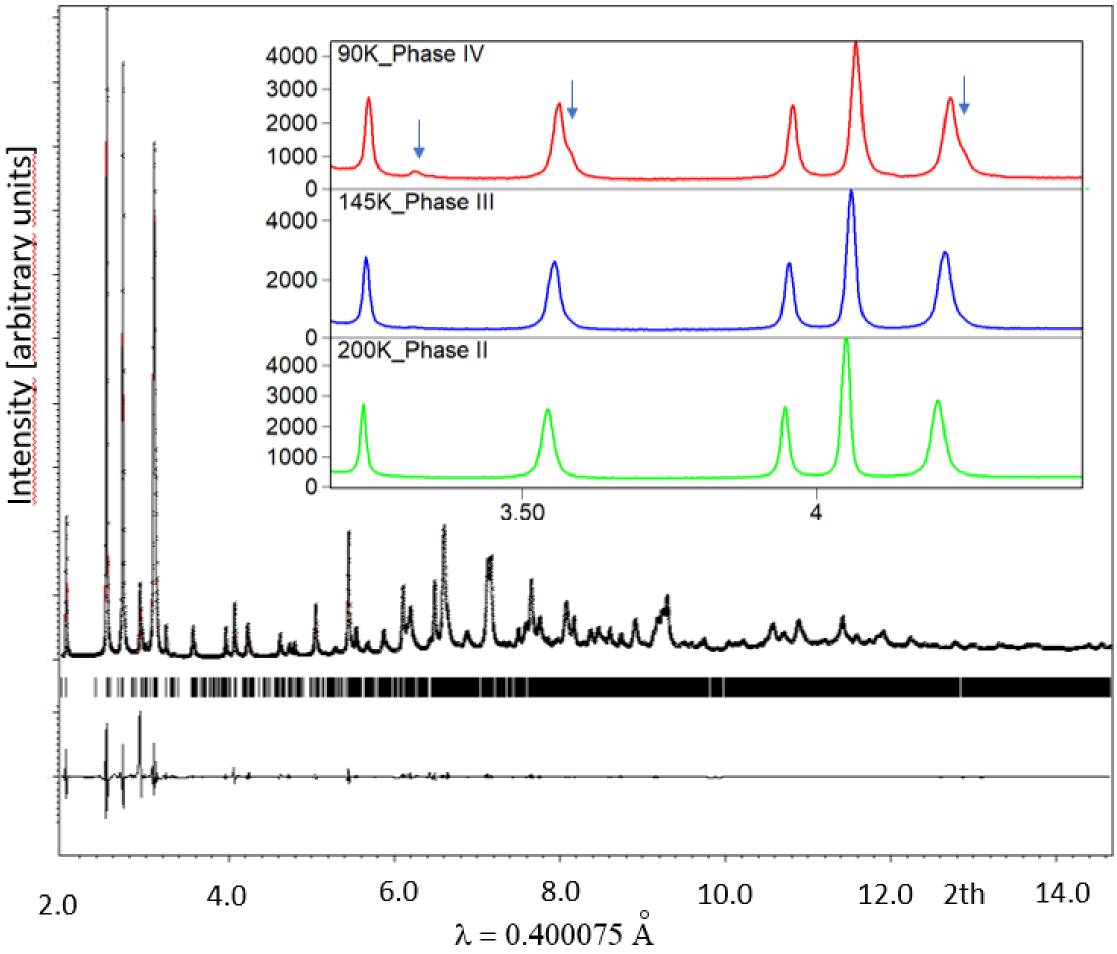


**Figure S1**. *Le Bail profile refinement results for SXRD data collected for* ***1*** *– (pyrrolidinium)*$_3$*[Bi*$_2$*I*$_9$*] at 90 K;* ***Phase IV****; a=8.6027(1)Å, b=18.1808(3)Å, c=45.14445(7)Å, alpha= 90.061(1), beta=90.878(2), gamma=90.378(2),* $R_p$*=0.031,* $wR_p$*=0.059. The inset shows the comparison between selected peaks of Phase II, III and IV. The arrows mark new intensities that appear in Phase IV.*

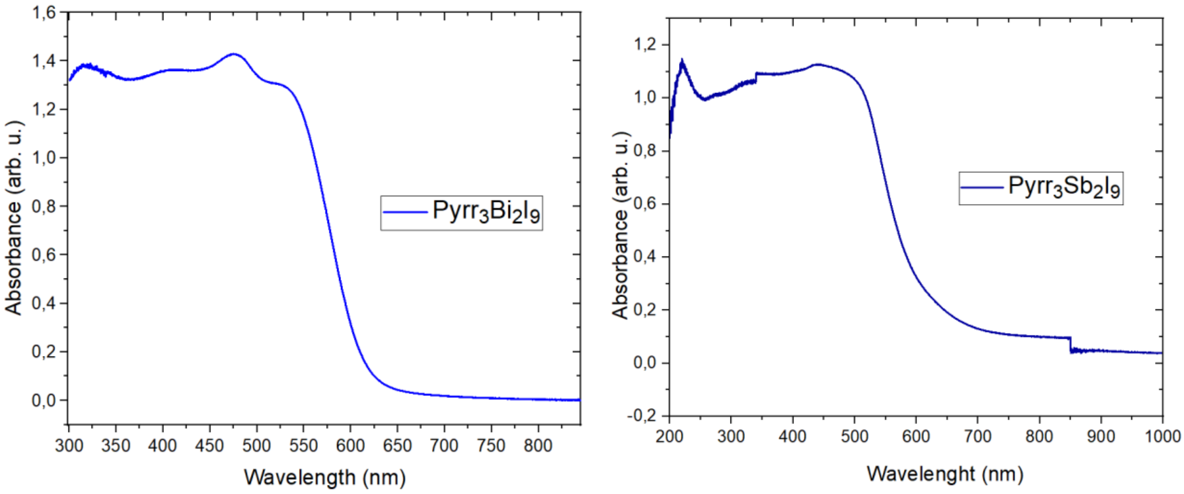


**Figure S2**. *Diffuse reflectance spectra of **1** (left) and **2** (right)*

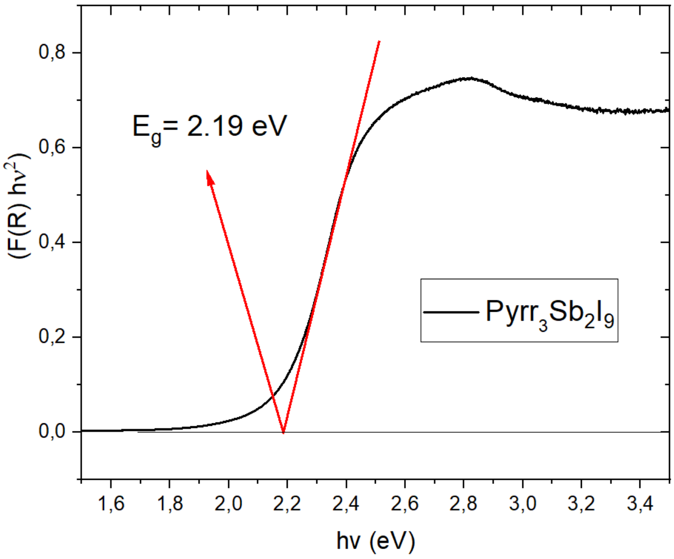


**Figure S3.** *Band gap (Eg) estimation for **2** with Tauc plot*

## Proton Magnetic Resonance ( $^{1}H$ NMR)

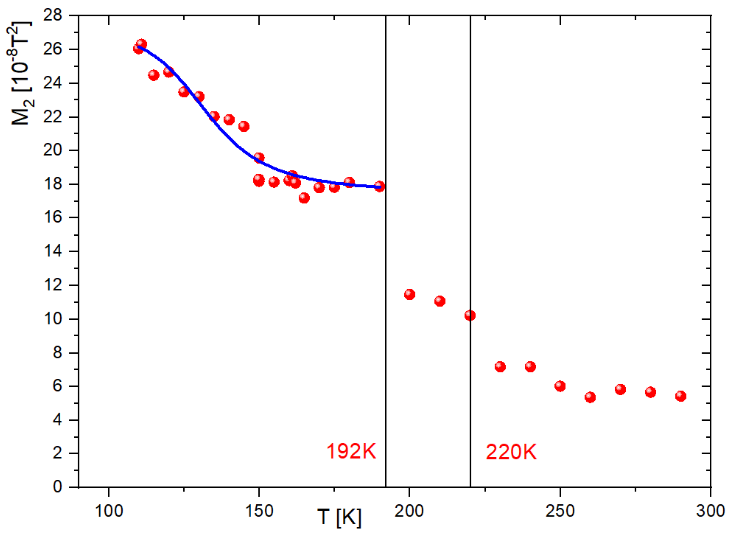


**Figure S4.** *$M_2$ Temperature dependence of second moment of $^1$H NMR line of* ***1***

Figure S4 depicts the temperature dependencies of the second moment $M_2$ of the $^1$H NMR line of **1**. The $M_2$ measurements reveal the detected phase transitions (PTs) observed through DSC and dielectric studies, as evidenced by a rapid reduction of the NMR line. The theoretical value of the second moment for a rigid lattice was calculated using the van Vleck formula [1]:

$$M_2 = \frac{3}{5} I(I+1)\gamma_H^2 \hbar^2 \frac{1}{N} \sum_{i=1}^{N} \sum_{j=1}^{N} R_{ij}^{-6} ,$$

where I represents the hydrogen nuclei spin, $\gamma_H$ denotes the gyromagnetic ratio of proton spin ($\gamma_H$= 26.75221 107 rad/Ts), Ri,j indicate the internuclear distance in the whole sample, and N refers to the number of resonant spins in the molecule. The computed value of the second moment for a rigid molecule ($M_{2rigid}$) was determined to be 22.14 × $10^{-8}$T$^2$, which is lower than the measured value. For temperatures up to the PT at 192 K, the reduction of the second moment can be analyzed based on the BPP formula [2]:

$$M_2 = M_2 + (M_2 + M_2)\tfrac{2}{\pi} arctg(\gamma_H \sqrt{M_2}\tau_c)$$

where the correlation time follows the Arrhenius law $\tau_c$= $\tau_o$ exp($E_a$/RT), $M_{2rigid}$ and $M_{2motion}$ represent the second moment values before (rigid) and after (motion) the onset of a given motion, respectively. Fitting the data yields the following parameters: $E_a$ = 10.1 kJ/mol and $\tau_{c0}$

= 8.04 $10^{-10}$ s (as shown by the fitting dotted curve in Fig. M2). Notably, in the vicinity of the PT at 192 K, a significant reduction of the second moment from 18 $10^{-8}T^2$ to 12 $10^{-8}T^2$ is observed, confirming a significant increase of the dynamics of the pyrrolidinium cations, the next drop is observed around 220 K. However, the value of M2~5.5 $10^{-8}T^2$ in the HT phase indicates that pyrrolidinium cations do not follow free rotations.

[1] J.H. Van Vleck, Phys. Rev., 1948, 74, 1168

[2] N. Bloembergen, E.M. Purcell, R.V. Pond, Phys. Rev., 1948, 73, 679
